# Supplementary material for: Bacterial Aspiration Pneumonia in Generalized Convulsive Status Epilepticus: Incidence, Associated Factors and Outcome
Source: J Clin Med. 2022 Nov 10;11(22):6673. doi: 10.3390/jcm11226673 (PMC9695142; doi:10.3390/jcm11226673)
Supplement: Supplementary file 1 [file jcm-11-06673-s001.zip › jcm-1930140-supplementary.pdf]

Journal of
Clinical Medicine

MDPI

Table S1. Comparison of patients with BAP according to amoxicillin/clavulanic acid susceptibility among causative pathogens.

**Characteristics**	**BAP (n=54)**		
	**≥1 micro-organism non-susceptible to ACA (n=13)**	**All micro-organisms susceptible to ACA (n=41)**	**p value**
**Demographics**			
Age (years)	64 (42-67)	55 (43-64)	0.728
Male sex	8 (62)	29 (71)	0.733*
mRS score 0-1	4 (31)	22 (54)	0.150
SAPS II (without age)	57 (26-64)	51 (40-56)	0.857
Immunosuppression	2 (15)	0 (0)	0.055*
Colonization with ESBL-producing bacterium	4 (31)	1 (2)	**0.010***
Antibiotics before ICU admission	4 (31)	0 (0)	**0.002***
**Outcomes**			
Mechanical ventilation duration (days)	2.06 (1.71-16.38)	4.04 (1.71-7.79)	0.838
ICU stay length (days)	6.67 (5.67-16.71)	7.00 (4.04-11.60)	0.413
ICU mortality	1 (8)	5 (12)	1.000*
3-month poor functional outcome	5 (38)	19 (50)	0.472
3-month mortality	2 (15)	7 (18)	1.000*

Categorical variables were expressed as number (percentage) and compared by a Chi-squared test or Fisher's exact test when specified by *. Continuous variables were expressed as median (inter-quartile range) and *t*-test was performed (Welch or Wilcoxon tests, as appropriate).
Missing values (non-susceptible to ACA; susceptible to ACA): 3-month outcomes, 4 (0;3).
Abbreviations: BAP, bacterial aspiration pneumonia; ACA, amoxicillin-clavulanic acid; ICU, intensive care unit.
